# Validation of the Mobile Application Rating Scale (MARS)

**DOI:** 10.1371/journal.pone.0241480

**Published:** 2020-11-02

**Authors:** Yannik Terhorst, Paula Philippi, Lasse B. Sander, Dana Schultchen, Sarah Paganini, Marco Bardus, Karla Santo, Johannes Knitza, Gustavo C. Machado, Stephanie Schoeppe, Natalie Bauereiß, Alexandra Portenhauser, Matthias Domhardt, Benjamin Walter, Martin Krusche, Harald Baumeister, Eva-Maria Messner

**Affiliations:** 1 Department of Research Methods, Institute of Psychology and Education, University Ulm, Ulm, Germany; 2 Department of Clinical Psychology and Psychotherapy, Institute of Psychology and Education, University Ulm, Ulm, Germany; 3 Department of Rehabilitation Psychology and Psychotherapy, Institute of Psychology, Albert-Ludwigs-University Freiburg, Freiburg im Breisgau, Germany; 4 Department of Clinical and Health Psychology, Institute of Psychology and Education, University Ulm, Ulm, Germany; 5 Department of Sport Psychology, Institute of Sports and Sport Science, University of Freiburg, Freiburg, Germany; 6 Department of Health Promotion and Community Health, Faculty of Health Sciences, American University of Beirut, Beirut, Lebanon; 7 Academic Research Organization, Hospital Israelita Albert Einstein, São Paulo, Brazil; 8 Westmead Applied Research Centre, Westmead Clinical School, Faculty of Medicine and Health, The University of Sydney, Sydney, Australia; 9 Cardiovascular Division, The George Institute for Global Health, Sydney, Australia; 10 Department of Internal Medicine 3 – Rheumatology and Immunology, University Hospital Erlangen, Friedrich-Alexander University Erlangen-Nuremberg, Erlangen, Germany; 11 Institute for Musculoskeletal Health, Sydney, New South Wales, Australia; 12 Sydney School of Public Health, Faculty of Medicine and Health, The University of Sydney, Sydney, New South Wales, Australia; 13 School of Health, Medical and Applied Sciences, Appleton Institute, Physical Activity Research Group, Central Queensland University, Rockhampton, Queensland, Australia; 14 Department of Internal Medicine I, Gastroenterology, University Hospital Ulm, Ulm, Germany; 15 Department of Rheumatology and Clinical Immunology, Charité – Universitätsmedizin Berlin, Berlin, Germany; Brown University, UNITED STATES

## Abstract

**Background:**

Mobile health apps (MHA) have the potential to improve health care. The commercial MHA market is rapidly growing, but the content and quality of available MHA are unknown. Instruments for the assessment of the quality and content of MHA are highly needed. The Mobile Application Rating Scale (MARS) is one of the most widely used tools to evaluate the quality of MHA. Only few validation studies investigated its metric quality. No study has evaluated the construct validity and concurrent validity.

**Objective:**

This study evaluates the construct validity, concurrent validity, reliability, and objectivity, of the MARS.

**Methods:**

Data was pooled from 15 international app quality reviews to evaluate the metric properties of the MARS. The MARS measures app quality across four dimensions: engagement, functionality, aesthetics and information quality. Construct validity was evaluated by assessing related competing confirmatory models by confirmatory factor analysis (CFA). Non-centrality (RMSEA), incremental (CFI, TLI) and residual (SRMR) fit indices were used to evaluate the goodness of fit. As a measure of concurrent validity, the correlations to another quality assessment tool (ENLIGHT) were investigated. Reliability was determined using Omega. Objectivity was assessed by intra-class correlation.

**Results:**

In total, MARS ratings from 1,299 MHA covering 15 different health domains were included. Confirmatory factor analysis confirmed a bifactor model with a general factor and a factor for each dimension (RMSEA = 0.074, TLI = 0.922, CFI = 0.940, SRMR = 0.059). Reliability was good to excellent (Omega 0.79 to 0.93). Objectivity was high (ICC = 0.82). MARS correlated with ENLIGHT (ps<.05).

**Conclusion:**

The metric evaluation of the MARS demonstrated its suitability for the quality assessment. As such, the MARS could be used to make the quality of MHA transparent to health care stakeholders and patients. Future studies could extend the present findings by investigating the re-test reliability and predictive validity of the MARS.

## Introduction

The global burden of disease is high across the world [1]. Mobile health applications (MHA) have the potential to substantially improve health care by providing accessible, effective, cost-efficient, and scalable interventions, as well as health information that can improve the screening, diagnostics, prevention and treatment of diseases [2–6].

Currently, there are over 300,000 MHA available in the app stores, and more than 200 MHA are added each day [7]. Several randomized controlled trials have shown that MHA can be an effective intervention tool for the prevention and treatment of various health conditions [6]. A recent meta-analysis of randomized trials reported small to moderate pooled effects of MHA for improving depression, anxiety, stress levels, and quality of life [6, 8]. However, the number of evidence-based MHA on the MHA market is surprisingly small [3, 4, 9, 10]. The lack of evidence-based MHA in combination with the rapidly growing MHA market highlight that patients and health care providers need better guidance to identify high-quality MHA that meet patients’ needs [11]. Reliable and valid measures to assess the quality of MHA are needed to provide such information to health care stakeholders and patients.

The Mobile Application Rating Scale (MARS) is the most widely used scale for evaluating the quality and content of MHA [3, 10, 12, 13–24]. The MARS is a multidimensional instrument to assess MHA quality and was developed based on semantic analysis and synthesis of relevant literature [16]. In total four separate dimensions were derived: engagement, functionality, aesthetics and information quality [16]. The original validation study showed good reliability of the subscales (α = 0.80 to 0.89) and the overall scale (α = 0.90), and good objectivity (subscales: Intra-class correlation (*ICC*) = 0.50 to 0.80; overall = 0.90) [16]. These results were replicated in several other studies investigating the metric basic of translated versions of the MARS [25–27]. However, the generalizability of previous findings is limited due to small sample sizes, and MHA used for specific health conditions and geographic areas. Furthermore, crucial metric properties have not been extensively evaluated: 1) no study has evaluated the construct validity of the MARS–meaning whether the proposed four separate dimensions are indeed independent—, 2) the concurrent validity with other quality instruments, such as the ENLIGHT instrument [28], is unknown, and 3) the findings regarding the concurrent validity with user-ratings in the app stores are inconclusive to this point [3, 14, 16]. Moreover, there are some methodological limitations in previous MARS evaluations (e.g., using Cronbach’s alpha for reliability [29–31]).

In an effort to address the aforementioned research gaps, this study aimed to validate the MARS based on pooled MARS data from 15 international reviews assessing the quality and content of MHA in various health conditions. The following research questions were investigated:

1. What is the validity of the MARS in terms of:
  - Construct validity: What is the latent structure of the MARS and are the proposed four dimensions independent?
  - Concurrent validity: What are the correlations between the MARS and another frequently used quality assessment tool called ENLIGHT [28]?
2. Reliability: What is the internal consistency of the overall MARS and its subscales?
3. Objectivity: What is the agreement between reviewers?

## Methods

### Study design

This is a validation study evaluating the metric quality of the MARS [16]. Similar to an individual patient data meta-analysis approach [32], research groups using the MARS were contacted and asked to provide their primary data (= quality ratings of MHA). Subsequently, all data sets provided were verified, homogenized, and merged into a single data set.

### Inclusion criteria and search

To obtain a large data set, only reviews about MHA using the MARS were eligible. Reviews that used the MARS to assess the quality of MHA were identified through literature searches conducted in Google Scholar and PubMed in July 2019, using terms such as MHA reviews, app quality or MARS. The literature searches were conducted by PP, YT and EM. The corresponding authors of the identified reviews were contacted and asked to share their data. Data from on-going reviews in which the authors were involved were also included. Data from the original validation study of the MARS [16] were excluded to obtain an independent sample for the present validation study.

### Measurement: Mobile Application Rating Scale

The MARS is a multidimensional instrument assessing the quality of MHA [16]. The quality assessment consists of a total of 19 items covering four dimensions. The dimensions are: (A) engagement (5 items: fun, interest, individual adaptability, interactivity, target group), (B) functionality (4 items: performance, usability, navigation, gestural design), (C) aesthetics (3 items: layout, graphics, visual appeal), and (D) information quality (7 items: accuracy of app description, goals, quality of information, quantity of information, quality of visual information, credibility, evidence base). All items are assessed on a 5-point scale (1-inadequate, 2-poor, 3-acceptable, 4-good, and 5-excellent). Items assessing information quality can also be rated as not applicable (e.g., in case of missing evidence or missing visual information).

### Statistical analysis

#### Validity

**Fig 1:**
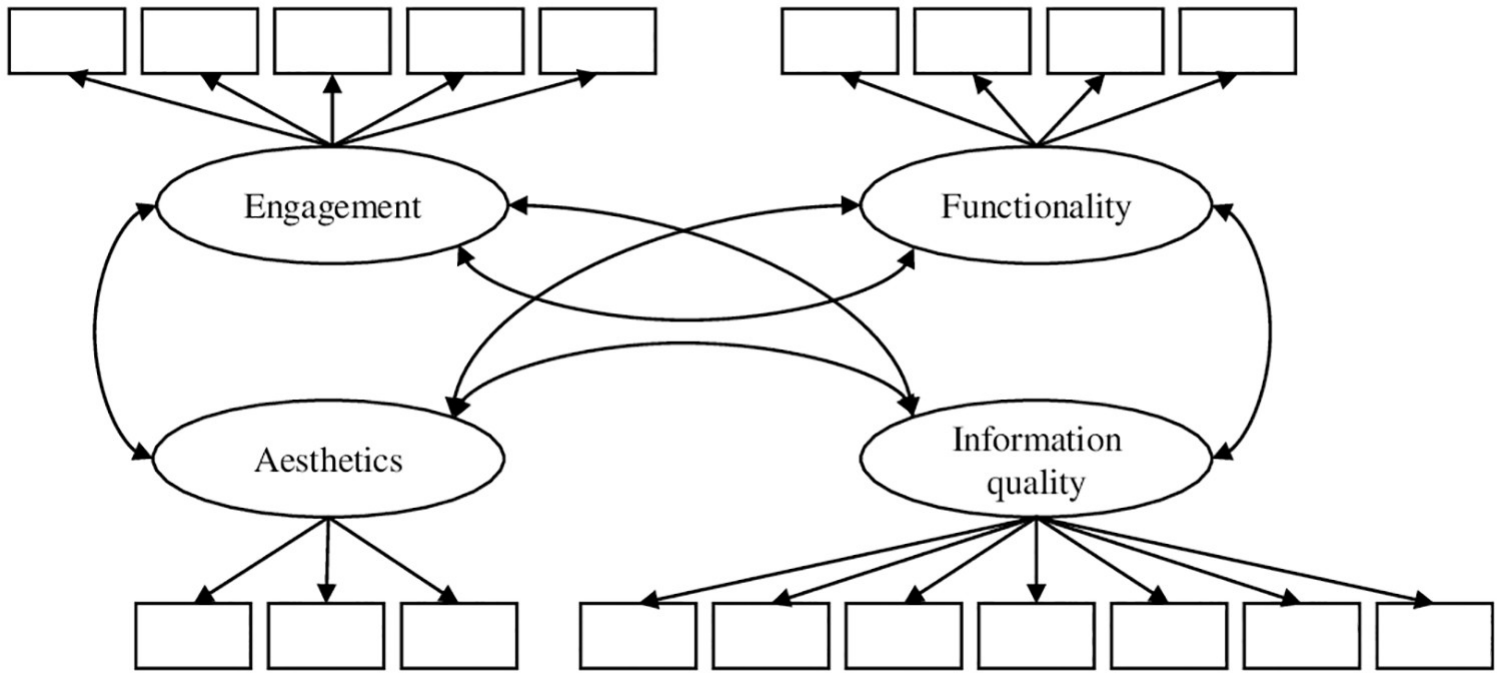
Hypothesized CFA model 1 of the MARS. Item-wise error variances are not represented in the models; correlations between errors were not allowed.

**Fig 2:**
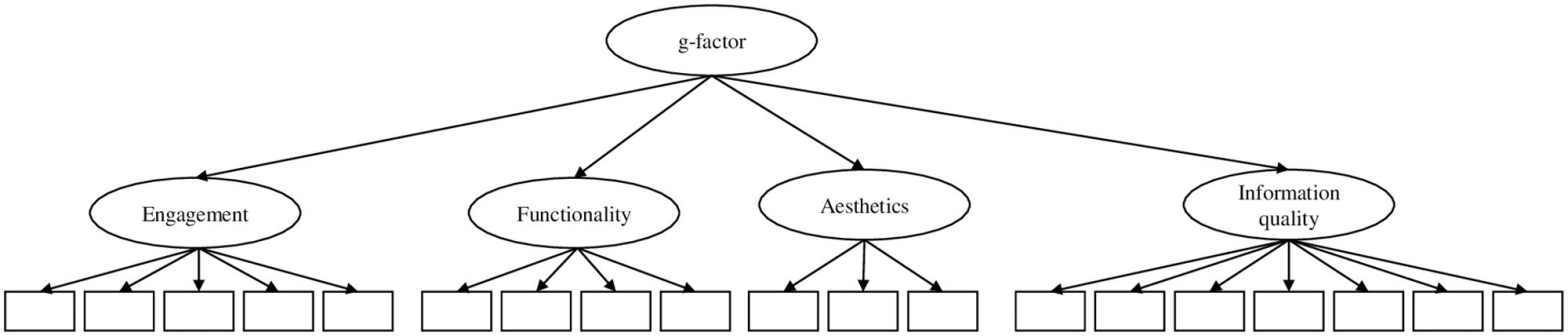
Hypothesized CFA model 2 of the MARS. Item-wise error variances are not represented in the models; correlations between errors were not allowed.

**Fig 3:**
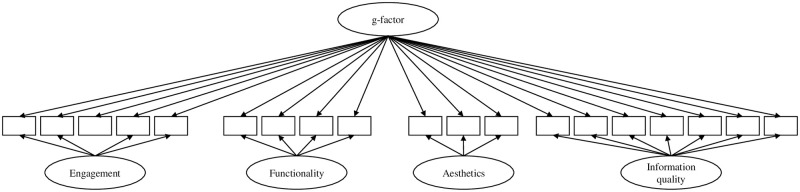
Hypothesized CFA model 3 of the MARS. Item-wise error variances are not represented in the models; correlations between errors were not allowed.

**Fig 4:**
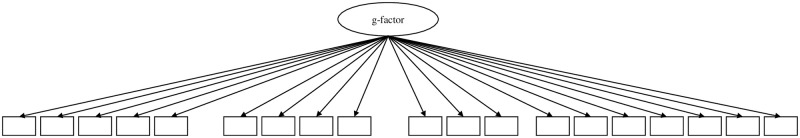
Hypothesized CFA model 4 of the MARS. Item-wise error variances are not represented in the models; correlations between errors were not allowed.

*Construct validity*: *Confirmatory factor analysis*. Confirmatory factor analysis (CFA) was applied to examine four proposed models. The MARS was designed to measure app quality. Based on the four subscales engagement, functionality, aesthetics, and information quality, we hypothesized four competing confirmatory models:

1. Model 1 consisted of four latent factors accounting for the item co-variance of the respective subscales, correlations between the four latent factors were allowed (see Fig 1);
2. Model 2 assumed a latent factor for the items of each subscale, and in contrast to model 1, a higher order factor was introduced to account for correlations between the factors (see Fig 2);
3. Model 3 has one general latent factor (g-factor) accounting for the co-variance of all items and four residual factors accounting for the remaining co-variances of the respective subscale items (see Fig 3);
4. Model 4 assumed only a general factor (see Fig 4).

Due to the high power of the χ2-test and its tendency to reject slightly mis-specified models [33–35], the model fit was evaluated using various fit indices: the root mean square error of approximation (RMSEA) as a non-centrality parameter, the standardized root mean square residual (SRMR) as a residual index, the confirmatory fit index (CFI) and the Tucker-Lewis index (TLI) as incremental indices. Cut-off values for an acceptable goodness of fit were based on standard modeling criteria: RMSEA < 0.06, SRMR < 0.08, CFI > 0.95 and TLI > 0.95 [36]. Akaike information criterion (AIC) and the Bayesian information criterion (BIC) were used for model comparisons.

Full information maximum likelihood was used as a robust estimator given its capability to handle missing data [37, 38]. Hubert-White robust standard errors were obtained [38]. Modification indices were used to further investigate the structure of the MARS and potential sources of ill fit [39].

*Concurrent validity*. Since the MARS was designed to measure app quality, it should be related closely to other app quality metrics. Some of the included data sets provided both ratings using the ENLIGHT instrument and the MARS. Similar to the MARS, the ENLIGHT is a quality assessment tool for MHA [28], which assesses app quality covering seven dimensions: a. usability (3 items), b. visual design (3 items), c. user engagement (5 items), d. content (4 items), e. therapeutic persuasiveness (7 items), f. therapeutic alliance (3 items), and g. general subjective evaluation (3 items). Items are rated from 1 (= very poor) to 5 (= very good). The intra-rater-reliability of the ENLIGHT (ICC = 0.77 to 0.98) and the internal consistency (α = 0.83 to 0.90) are excellent [28].

Correlations were used to determine the concurrent validity between the MARS and ENLIGHT. All correlations reported in this study were calculated using correlation coefficient *r*, which ranges between 0 (no relationship) to 1 (perfect relationship) or -1 (perfect negative relationship) respectively. For all correlation analyses, the alpha-level was 5%. P-values were adjusted for multiple testing using the procedure proposed by Holm [40].

#### Reliability: Internal consistency

As a variant of reliability, internal consistency was determined. Omega was used as reliability coefficient [41]. Compared to the widely used Cronbach’s Alpha, Omega provides a more unbiased estimation of reliability [29–31]. The procedures introduced by Zhang and Yuan [42] were used to obtain robust coefficients and bootstrapped bias-corrected confidence intervals. A reliability coefficient of < 0.50 was considered to be unacceptable, 0.51–0.59 to be poor, 0.60–0.69 to be questionable, 0.70–0.79 to be acceptable, 0.80–0.89 to be good, and > 0.90 to be excellent [43].

#### Objectivity: Intra-class correlation

The MARS comes with a standardized online training for reviewers [16]. Following the training, the MARS assessment is suggested to be either conducted by a single rater or by two raters (pooling their ratings) [16]. Consistency between raters was examined by calculating intra-class correlation based on a two-way mixed-effects model [44]. A cut-off of ICC above 0.75 (Fleiss, 1999) was used to define a satisfactory inter-rater agreement. All data sets based on ratings of two reviewers were included in this analysis.

#### Analysis software

The software R was used for all analyses [45], except for the intra-class correlation, which was calculated using SPSS 24 [46]. For the CFA, the R package “lavaan” (version: 0.5–23.1097) was deployed [47]. Omega was assessed using “coefficient alpha” [42]. Correlations were calculated using “psych” (version: 1.7.8.) [48].

## Results

### Sample characteristics

The literature searches identified a total of 18 international reviews that assessed the quality of MHA using the MARS. All research groups that have published an eligible review were contacted. In total, 15 of the 18 contacted research groups responded and agreed to share their data [3, 10, 12, 14, 15, 18, 19, 22, 24, 49–54]. The present sample consists of N = 1299 MHA. MHA targeting physical, mental and behavioral health, as well as specific target groups were included: anxiety (n = 104), low back pain (n = 58), cancer (n = 78), depression (n = 38), diet (n = 25), elderly (n = 84), gastrointestinal diseases (n = 140), medication adherence (n = 9), mindfulness (n = 103), pain (n = 147), physical activity (n = 312), post-traumatic stress disorder (n = 87), rheumatism (n = 32), weight management (n = 66), and internalizing disorder MHA for children and youth (n = 16). For all included data sets, the MARS rating was conducted by researchers holding at least a B.Sc. degree.

**Fig 5:**
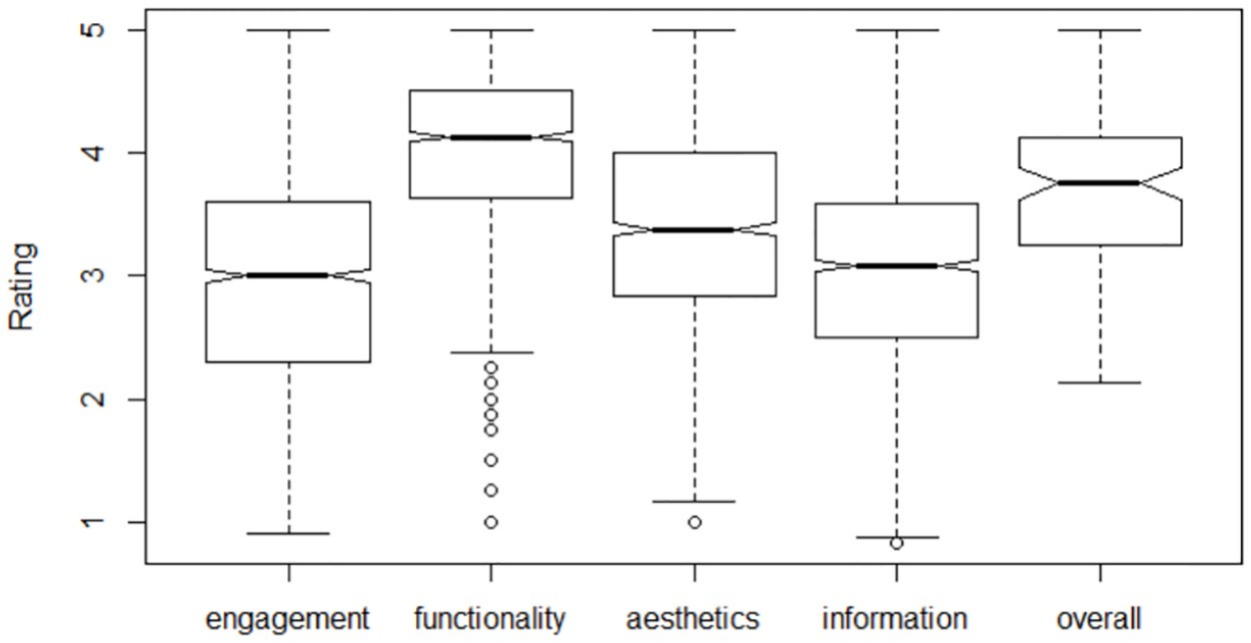
Quality of included MHA.

The overall quality of these MHA based on the quality assessment using MARS was moderate (mean MARS score [M] = 3.74, standard deviation [SD] = 0.59). The quality of MHAs was highest in relation to the functionality dimension (M = 4.03, SD = 0.67), followed by aesthetics (M = 3.40, SD = 0.87), information quality (M = 3.06, SD = 0.72) and engagement (M = 2.96, SD = 0.90) (see Fig 5).

The MARS assesses the evidence base of an app using the question “Has the app been trialled/tested; must be verified by evidence (in published scientific literature)?”. Overall, 1230 (94.8%) of all included MHAs were rated as not evidence-based.

### Construct validity: Confirmatory factor analysis

**Fig 6:**
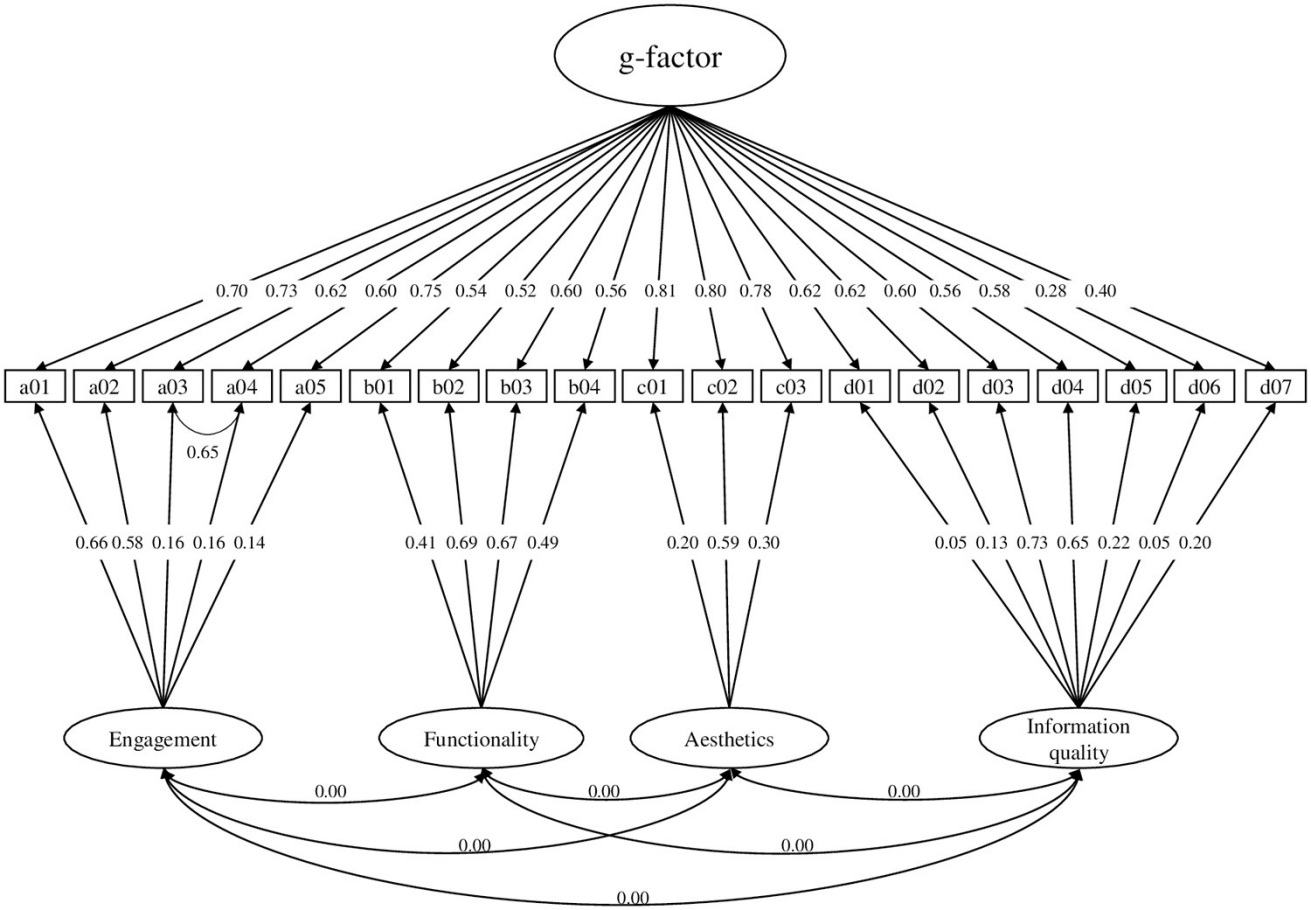
Model 3a. Loadings are standardized; correlations between all latent variables were set to zero; item-wise error variances have been excluded; Model 3a differs from the a-priori defined model 3 in the correlation between item 3 (a03) and item 4 (a04).

None of the a-priori defined confirmatory models were confirmed by CFA. The best-fitting model was model 3. Model 3 was further investigated using modification indices. Introducing a correlation between items 3 and 4 (= Model 3a) yielded an acceptable model fit. Fit indices of all models are presented in Table 1. Model 3a is presented in Fig 6.

**Table 1:** Model fit.

Model	AIC	BIC	RMSEA	SRMR	TLI	CFI
Model 1	49110	49437	0.110 (0.106 to 0.113)	0.095	0.814	0.841
Model 2	49182	49497	0.115 (0.111 to 0.119)	0.098	0.811	0.837
Model 3	48132	48525	0.093 (0.088 to 0.097)	0.095	0.878	0.905
3a	47589	47987	0.074 (0.070 to 0.078)	0.059	0.922	0.940
Model 4	52102	52397	0.166 (0.162 to 0.170)	0.099	0.605	0.649

Note: AIC: Akaike information criterion; BIC: Bayesian information criterion; RMSEA: root mean square error of approximation (RMSEA); SRMR: standardized root mean square residual; CFI: the confirmatory fit index; TLI: Tucker-Lewis index.

### Concurrent validity

A total of 120 MHA were rated using both the ENLIGHT instrument and the MARS. Correlations between MARS and ENLIGHT were calculated based on the respective subsample. Correlations are presented in Table 2.

**Table 2:** Correlations between the MARS and ENLIGHT using a subsample of apps.

	MARS: Engagement	MARS: Functionality	MARS: Aesthetics	MARS: Information	MARS: Overall
ENLIGHT (n = 120)	*r*^a^	*r*^a^	*r*^a^	*r*^a^	*r*^a^
Usability	0.51***	0.80***	0.68***	0.39***	0.71***
Design	0.63***	0.66***	0.87***	0.57***	0.84***
Engagement	0.83***	0.52***	0.68***	0.47***	0.78***
Content	0.71***	0.54***	0.72***	0.68***	0.82***
Therapeutic persuasiveness	0.74***	0.42***	0.63***	0.54***	0.73***
Therapeutic alliance	0.56***	0.37***	0.44***	0.48***	0.58***
General subjective quality	0.69***	0.53***	0.68***	0.50***	0.74***
overall	0.83***	0.65***	0.81***	0.64***	0.91***

Note:

^a)^ correlation coefficient *r*, which ranges between 0 (no relationship) to 1 (perfect relationship) or -1 (perfect negative relationship) respectively.

* *P* < = 0.05,

** *P* < = 0.01,

*** *P* < = 0.001.

### Reliability: Internal consistency

The internal consistency of all sections was good to excellent (see Table 3).

**Table 3:** Internal consistency of the MARS.

Section	Reliability: Omega (CI)
A: Engagement	0.867 (0.853 to 0.880)
B: Functionality	0.871 (0.856 to 0.886)
C: Aesthetics	0.904 (0.895 to 0.913)
D: Information quality^1^	0.793 (0.773 to 0.813)
Overall^1^	0.929 (0.923 to 0.934)

Note:

^1)^ Item 19 was excluded due to high amount of missingness (95%), as it is rated NA (not applicable) if no evaluation is present.

### Objectivity: Intra-class correlation

To calculate the agreement of raters only data sets providing ratings of both reviewers were used. A total of 793 apps (= 15067 rated items per reviewer) were included in the intra-class correlation analysis. Overall, intra-class correlation was good: ICC = 0.816 (95% CI: 0.810 to 0.822). Section-wise ICC is summarized in Table 4.

**Table 4:** Objectivity of the MARS.

Section	Objectivity: ICC (95% CI)^a^
A: Engagement	0.790 (0.776 to 0.803)
B: Functionality	0.758 (0.740 to 0.774)
C: Aesthetics	0.769 (0.750 to 0.787)
D: Information quality	0.848 (0.839 to 0.857)
Overall	0.816 (0.810 to 0.822)

Note:

^a)^ Two-way mixed intra-class correlation coefficient (ICC) with 95% confidence intervals (CI).

## Discussion

To our knowledge, the present study is the first study to evaluate the construct validity of the MARS. Furthermore, this study builds on previous metric evaluations of the MARS [16, 25–27] by investigating its validity, reliability, and objectivity using a large sample of MHAs covering multiple health conditions. The CFA confirmed a bi-factor model consisting of a general g-factor and uncorrelated factors for each dimension of the MARS. Given the theoretical background of the MARS, the latent g-factor could represent a general quality factor or a factor accounting for shared variance introduced by the assessment methodology. Either way, the four uncorrelated factors confirm the proposed dimensions of the MARS [16]. Thus, the interpretation of the sum score for each dimension seems legit. However, the present analysis highlights that not all items are equally good indicators for the dimensions. Hence, a weighted average of the respective items of each of the four dimensions a) engagement, b) functionality, c) aesthetics and d) information quality would be more adequate.

Besides the construct validity, the concurrent validity was evaluated. High correlations to the ENLIGHT indicated a good concurrent validity. Furthermore, previous metric evaluations in terms of reliability and objectivity [16, 25–27] were replicated with the present MHA sample. Our findings showed that both reliability and objectivity of the MARS were good to excellent. Overall, considering the validity, reliability and objectivity results the MARS seems to be an app quality assessment tool of high metric quality.

The correlation between the MARS and the ENLIGHT instrument was high, at least in a sub-sample of the analyzed apps. This indicates good concurrent validity between both expert assessments. However, ENLIGHT contains a section assessing therapeutic alliance [28] which was only moderately covered by the MARS. The integration of therapeutic alliance in the MARS could further strengthen the quality of the MHA assessment. Especially in the context of conventional and digitalized health care, therapeutic alliance, guidance, and therapeutic persuasiveness, are important aspects along with persuasive design [25, 28, 55, 56].

Pooling data from multiple international reviews of the quality of MHA using MARS also provided an insight into the quality of many commercial MHA. While most MHA show high quality in terms of functionality and aesthetics, the engagement and information quality of MHA show high heterogeneity and an overall moderate quality. However, most striking is the lack of evidence-based MHA. Only 5% of the MHA were evaluated in studies (e.g., feasibility, uncontrolled longitudinal designs, RCT). This lack of evidence is in line with previous research and a major constraint in the secondary health market [3, 4, 9]. Creating an evidence-based MHA market and addressing central issues, like 1) data safety and privacy, 2) user adherence and 3) data integration, are core challenges that have to be solved to utilize the potential benefits of MHA in health care [57–59]. Using the MARS to make those issues transparent to health care stakeholders and patients, as well as establishing guidelines for the developments of MHA are both necessary and promising steps to achieve this goal [16, 57].

### Limitations

Some limitations of this study need to be noted. First, the main aim of this study was to evaluate the construct validity of the MARS. By including ratings of multiple reviewers across the world and multiple health conditions, we regard the external validity of the results as high. Nonetheless, the results might be only valid in the present sample and not transferable to other conditions, target groups or rating teams. Thus, the confirmed bifactor model should be challenged in other health conditions and also non-health apps. Notably, the necessary modification to the a-priori defined bifactor model should be closely investigated, since it was introduced based on modification indices and is of an exploratory nature. Second, the evaluation of the construct validity of the MARS might be biased due to the format of the MARS, as throughout the MARS all items are assessed on a 5-point scale. Since there is no variation in the item format, item-class specific variance cannot be controlled in the present evaluation. As a result, item-class variance might be attributed to the quality factor. These issues could be addressed in future studies by using a different item format. Also using a multi-method approaches, for example by integrating alternative assessments like the user version of the MARS [60] or the ENLIGHT [28] could lead to a more comprehensive assessment of the quality of MHA. Third, although reliability of the MARS was also a focus in this study (i.e., internal consistency), there are facets of reliability which are still unexplored. For instance, re-test reliability of the MARS has never been evaluated. To investigate re-test reliability, an adequate study design with time-shifted assessments of the same version of apps by the same reviewers is needed. This remains to be investigated in future studies. Finally, throughout the study, quality is discussed as a fundamental requirement for apps. However, the internal validity in the sense whether quality is predictive, for example, for engagement, adherence, effectiveness was not evaluated in this study. No study has yet investigated this using the MARS. Baumel and Yom-Tov [61] examined which design aspects are essential using the ENLIGHT instrument. For instance, engagement and therapeutic persuasiveness were identified as crucial quality aspects associated with user adherence [61]. Based on the high correlation between MARS and ENLIGHT, one could assume that their findings could also be applied to the MARS. However, this has to be confirmed in future studies. The role of quality should also be investigated in a more holistic model containing MHA specific features (e.g., persuasive design) [62, 63], user features (e.g., personality) and incorporating existing model such as the unified theory of acceptance and use of technology (UTAUT) [64].

## Conclusion

The MARS is a metrically well-suited instrument to assess MHA quality. Given the rapidly growing app market, scalable solutions to make content and quality of MHA more transparent to users and health care stakeholders are highly needed. The MARS may become a crucial part of such solutions. Future studies could extend the present findings by investigating the re-test reliability and predictive validity of the MARS.

## Supporting information

S1 Dataset (XLSX)

## Abbreviations

AIC: Akaike information criterion
BIC: Bayesian information criterion
CFA: Confirmatory factor analysis
CFI: confirmatory fit index
CI: confidence interval
ICC: intra-class correlation coefficient
JMIR: Journal of Medical Internet Research
M: Mean
MARS: Mobile Application Rating Scale
MHA: Mobile health app
*r*: correlation
RCT: randomized controlled trial
RMSEA: root mean square error of approximation
SD: Standard deviations
SRMR: standardized root mean square residual
TLI: Tucker-Lewis index
UTAUT: unified theory of acceptance and use of technology
α: Cronbach’s alpha
